# Osteoid Osteoma of the Distal Phalanx: A Rare Condition

**DOI:** 10.7759/cureus.19077

**Published:** 2021-10-27

**Authors:** Jimmy C Daher, Mohammad O Boushnak, Elie N Al Najjar, Esther H Tannoury, Ramzi C Moucharafieh

**Affiliations:** 1 Orthopedic Surgery, Lebanese American University Medical Center, Beirut, LBN; 2 Orthopedic Surgery, Lebanese University Faculty of Medicine, Beirut, LBN; 3 Radiology, Lebanese American University Medical Center, Beirut, LBN; 4 Orthopedics and Traumatology, Clemenceau Medical Center, Beirut, LBN; 5 Orthopedics and Traumatology, Saint George Hospital University Medical Center, Beirut, LBN

**Keywords:** surgical excision, osteoid osteoma, tumor, index finger, hand

## Abstract

Osteoid osteoma of the distal phalanges in the hand is rare and difficult to diagnose. We report a case of a 37-year-old Caucasian female patient who presented with a mass on the distal phalanx of the index finger. The patient was suffering from intermittent nocturnal pain for more than 18 months along with thickening, localized swelling, and clubbing of the distal phalanx of the right index finger. Radiographs revealed a lytic lesion of the distal phalanx of the right index finger with surrounding sclerosis. An MRI showed an intramedullary lesion with infiltration of the bone marrow, cortex, and surrounding tissue with focal sclerosis and elements of enhancements. A presumptive diagnosis of osteoid osteoma was made and surgical removal of the lesion by curettage and bone grafting was the treatment of choice. The curetted specimen was sent to pathology and the diagnosis of osteoid osteoma was confirmed. The patient was asymptomatic at six months postoperatively. Osteoid osteoma should be included in every differential diagnosis for patients presenting with atypical features of the distal phalanx of the hand.

## Introduction

Osteoid osteoma is a relatively frequent benign bone-forming tumor with an incidence of 11% of all benign tumors [1]. It mainly affects young male adults in the second and third decades of life, and frequently involves long bones of the lower extremity (50% of cases), predominantly the femur and the tibia [1]. In general, osteoid osteoma presents unique clinical, radiological, and physiological characteristics, which make it in many cases easy to diagnose. The night pain pattern, which is relieved by aspirin, is typical [1]. Radiologically, osteoid osteoma is a spherical tumor with a diameter less than 1.5 cm and a central zone of atypical bone termed “nidus” [2]. Pain is the result of an overproduction of prostaglandins by the tumor nidus at levels 100 to 1000 times that of normal tissue [3]. They induce vasodilation and resultant increased capillary permeability in the tissues surrounding the lesion and are believed to mediate tumor-related pain [2,4]. The rarity of osteoid osteomas in the hand (5-15% of osteoid osteomas) and the absence of its classical pattern make it extremely challenging to diagnose. Around 85% of these cases are initially misdiagnosed [5]. Moreover, osteoid osteoma of the phalanges is confined to a small area bordered by important structures such as the joint line, ligaments, growth plate, and nail bed. As such, it presents an array of atypical symptoms such as pulp swelling and nail clubbing, digit thickening, and other generalized signs of inflammation that might be attributed to various common etiologies (trauma, cyanosis, arthritis, or infection) [5]. In this case report, we present a rare case of osteoid osteoma involving the distal phalanx of the index finger of the right hand.

## Case presentation

**Figure 1:**
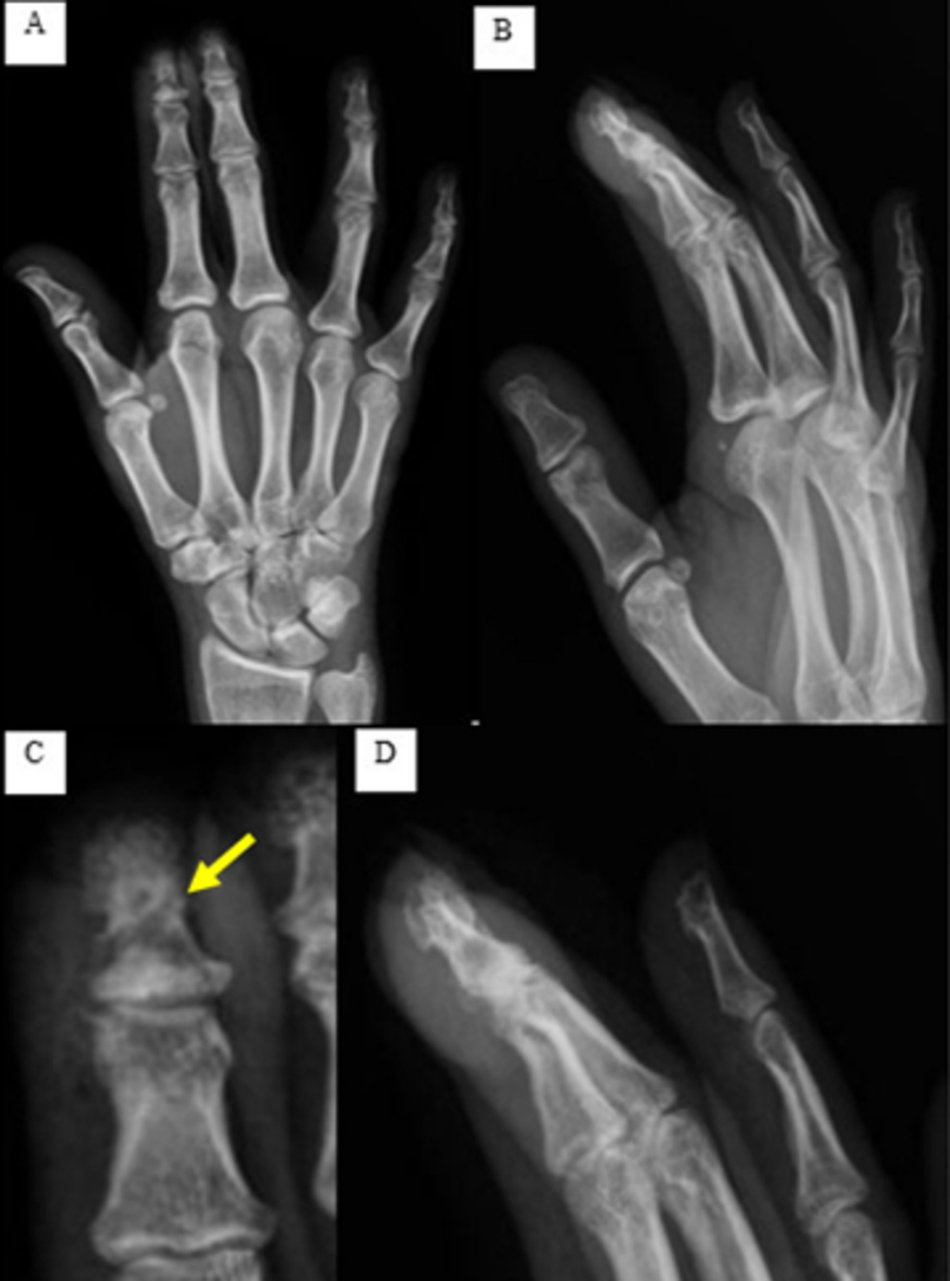
Preoperative radiographs of the right hand. (A) AP view; (B) lateral view; (C) and (D) AP and lateral magnified images, respectively, focusing on the distal phalanx of the index finger showing a lytic lesion of the distal phalanx with surrounding sclerosis (yellow arrow). AP, anteroposterior.

A 37-year-old right-handed Caucasian female patient presented to the outpatient orthopedic clinic with thickening of the distal phalanx of the right index finger that gradually increased over 18 months with intermittent pain that exacerbates at night, responding well to non-steroidal anti-inflammatory drugs (NSAIDs). On physical examination, localized swelling with clubbing of the distal part of the right index finger was noted, with both the pulp and the nail plate being enlarged and the nail fold angle disrupted. A slightly decreased range of motion in the distal interphalangeal joint was documented when compared to the contralateral hand. The patient denied any history of trauma or infection, and her blood profile for rheumatic and inflammatory disorders was normal as per the previous visit assessment in another facility. Radiographs performed revealed a lytic lesion of the distal phalanx of the right index finger with surrounding sclerosis (Figure 1).

**Figure 2:**
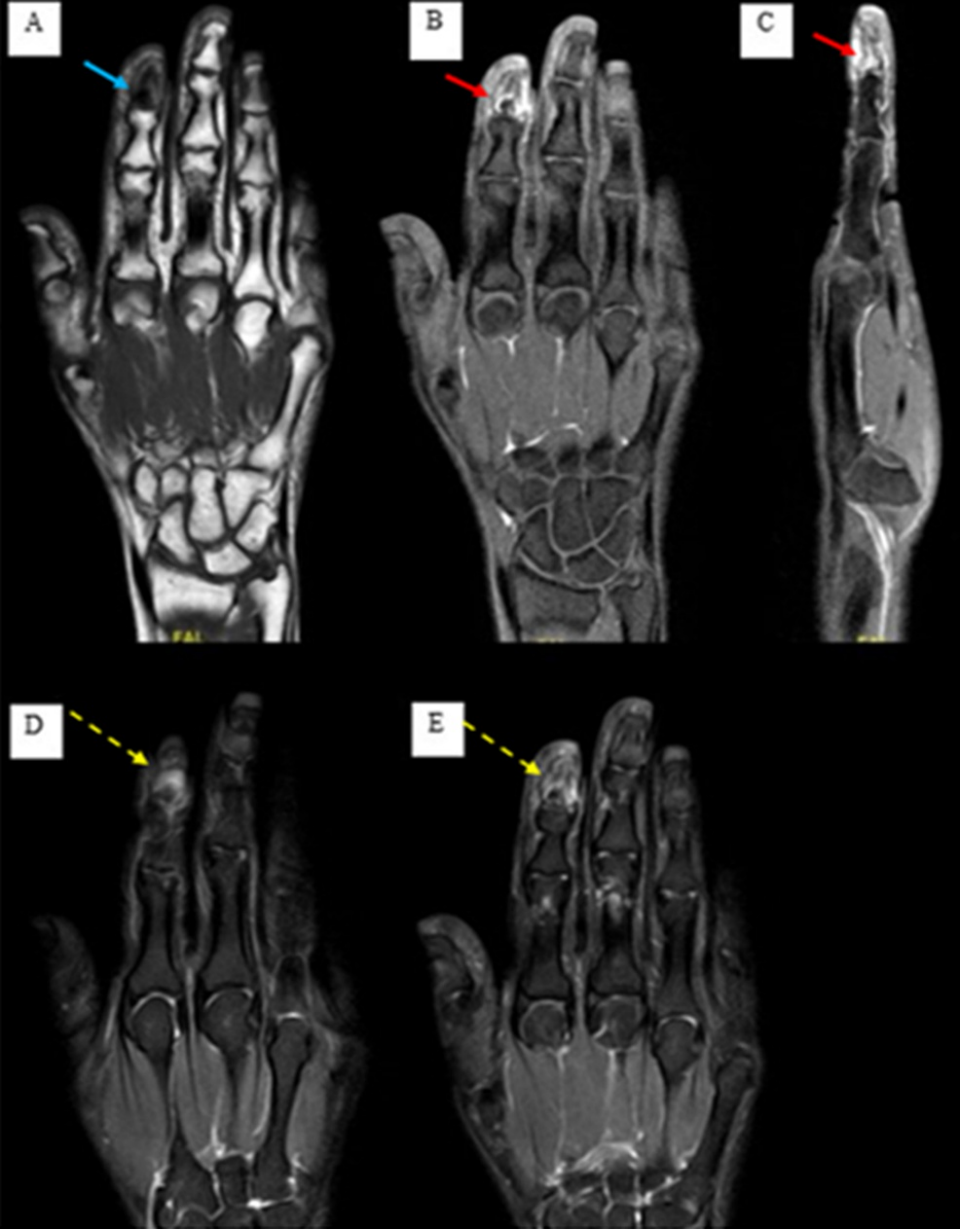
Preoperative MRI of the right hand showing an intramedullary lesion of the distal phalanx of the index finger with infiltration of the bone marrow, cortex, and surrounding tissue with focal sclerosis and elements of enhancement. (A) Hypo-intense on T1-weighted images (blue arrow) showing post-contract enhancement (B and C) (red arrows). Hyper-intense on T2 fat sat weighted images (D and E) (yellow arrows).

Magnetic resonance imaging (MRI) was done, which showed an intramedullary lesion of the index finger distal phalanx with infiltration of the bone marrow, cortex, and surrounding tissue with focal sclerosis and elements of enhancements (Figure 2).

**Figure 3:**
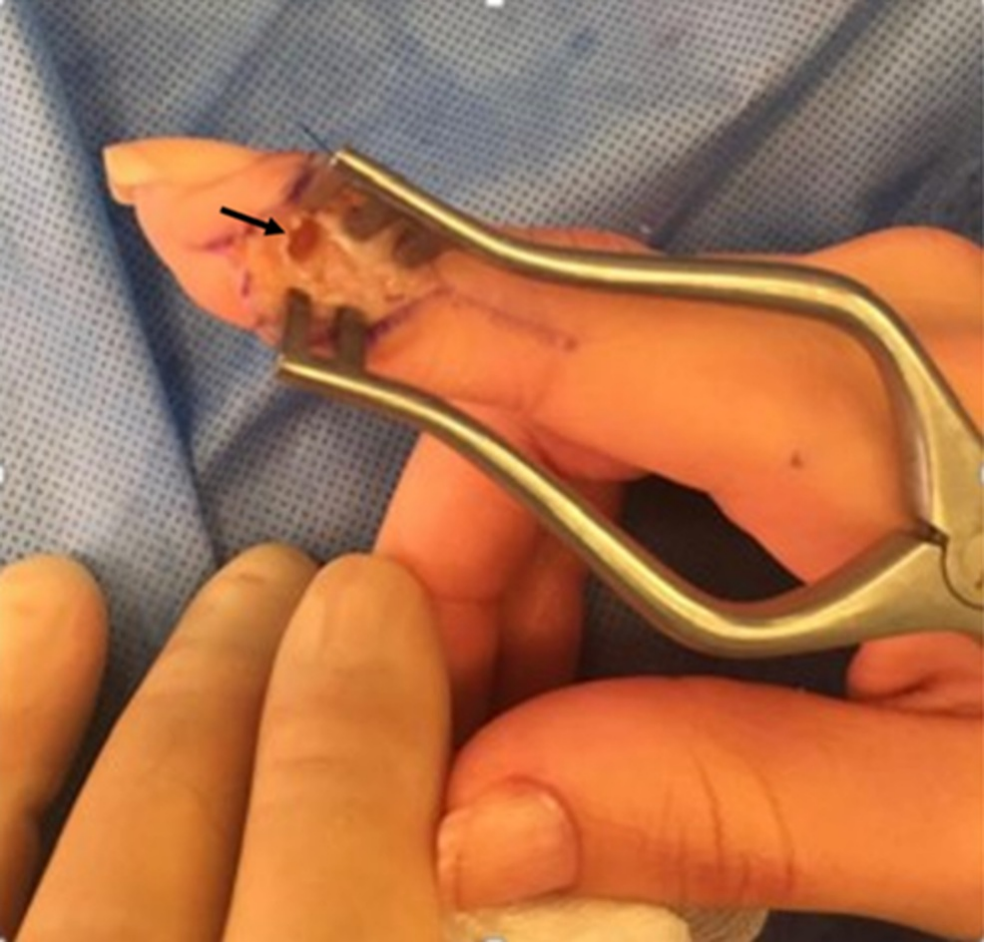
Intraoperative view of the right-hand distal phalanx of the index finger post curettage of nidus and drilling without perforation of the cortex (black arrow).

Based on clinical and radiographic evidence, a presumptive diagnosis of osteoid osteoma was made; however, other conditions including infection, glomus tumor, and blastoma were to be ruled out. The patient underwent surgery. Using a lateral approach, the terminal phalanx bone was exposed and the lateral cortex perforated. Curettage of the nidus was made with a dental burr and drill followed by removal of the sclerotic edges without further perforation of the cortex (Figure 3).

**Figure 4:**
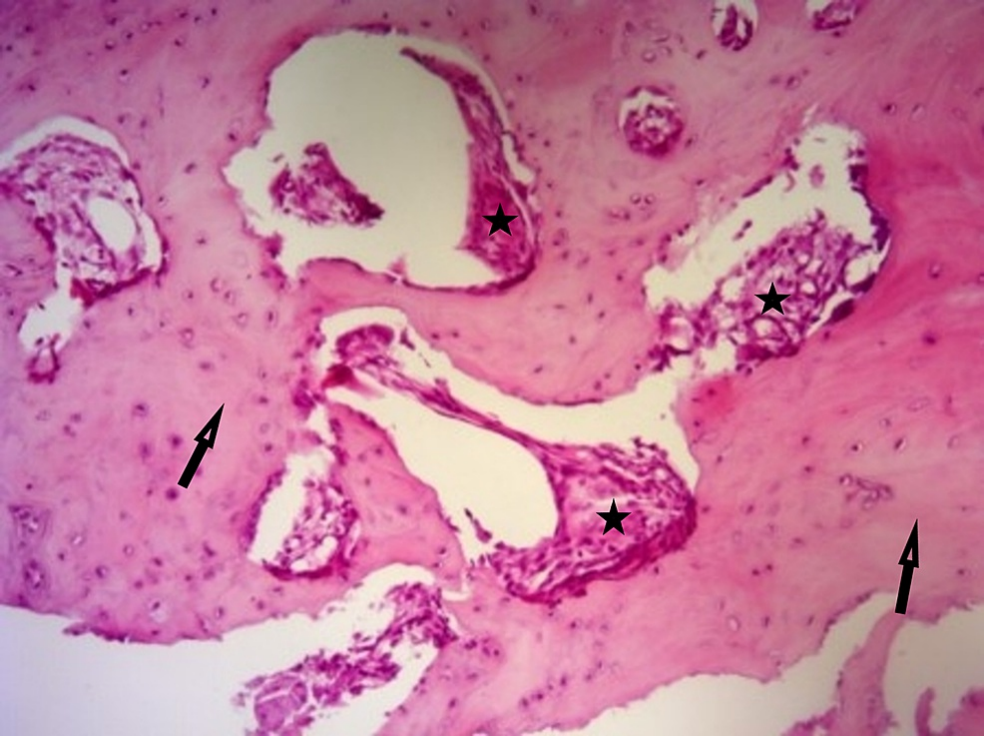
Post-operative pathological findings. Hematoxylin and eosin stain (100x) showing sclerotic bone (arrows) with focal fibrovascular tissue (stars).

Using fluoroscopic guidance, verification of complete evacuation was done, and bone grafting using a demineralized bone matrix was performed. The curetted specimen was sent to pathology. Postoperatively, the finger was immobilized with a splint and the patient reported immediate pain relief after a few days. Histopathological examination confirmed the diagnosis of osteoid osteoma (Figure 4).

**Figure 5:**
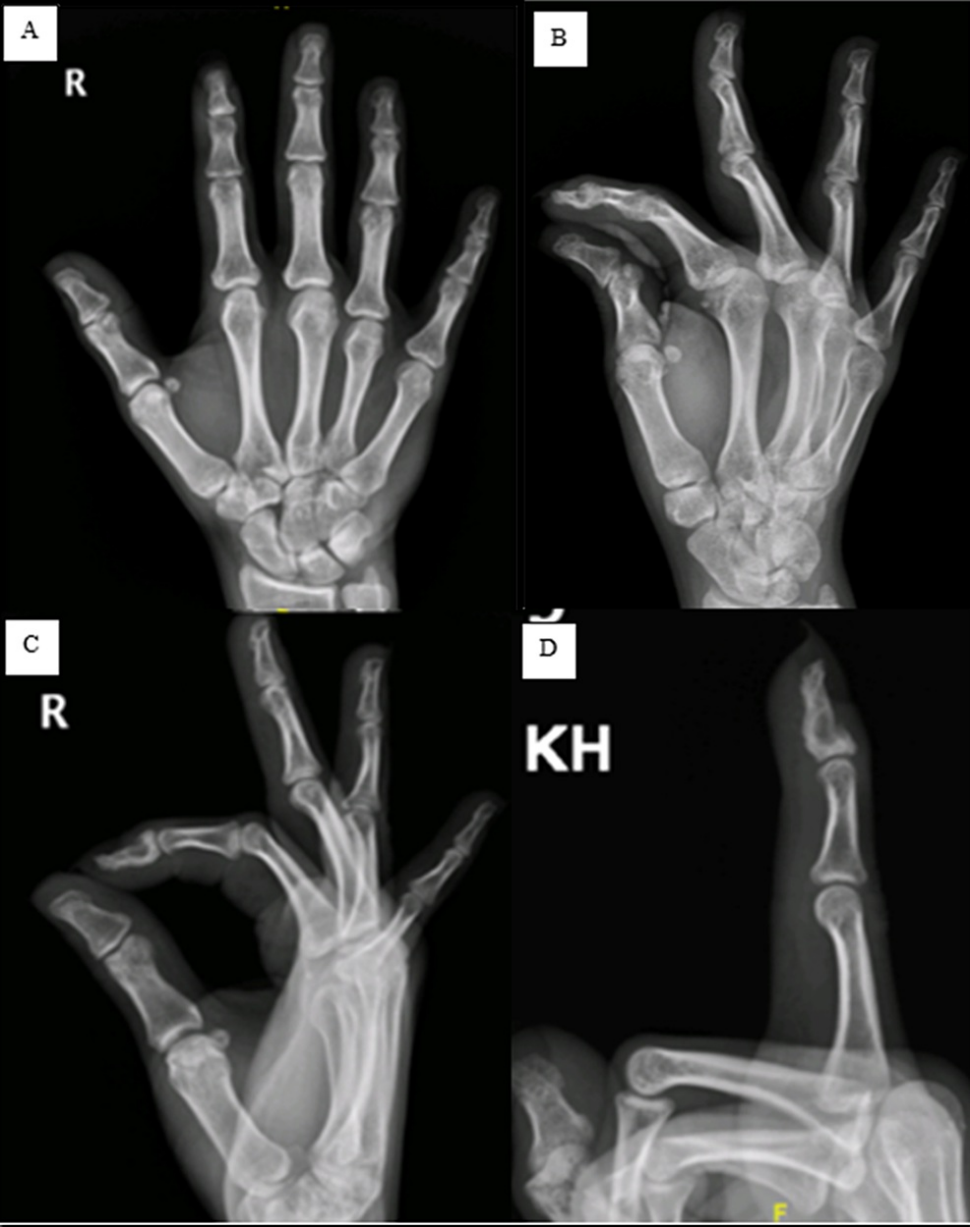
Follow-up radiographs at three months post-operative of the right-hand distal phalanx of the index finger showing no signs of fracture on (A) AP view, (B) oblique view, (C) lateral view, and (D) magnified lateral view focusing on the index finger. AP, anteroposterior.

**Figure 6:**
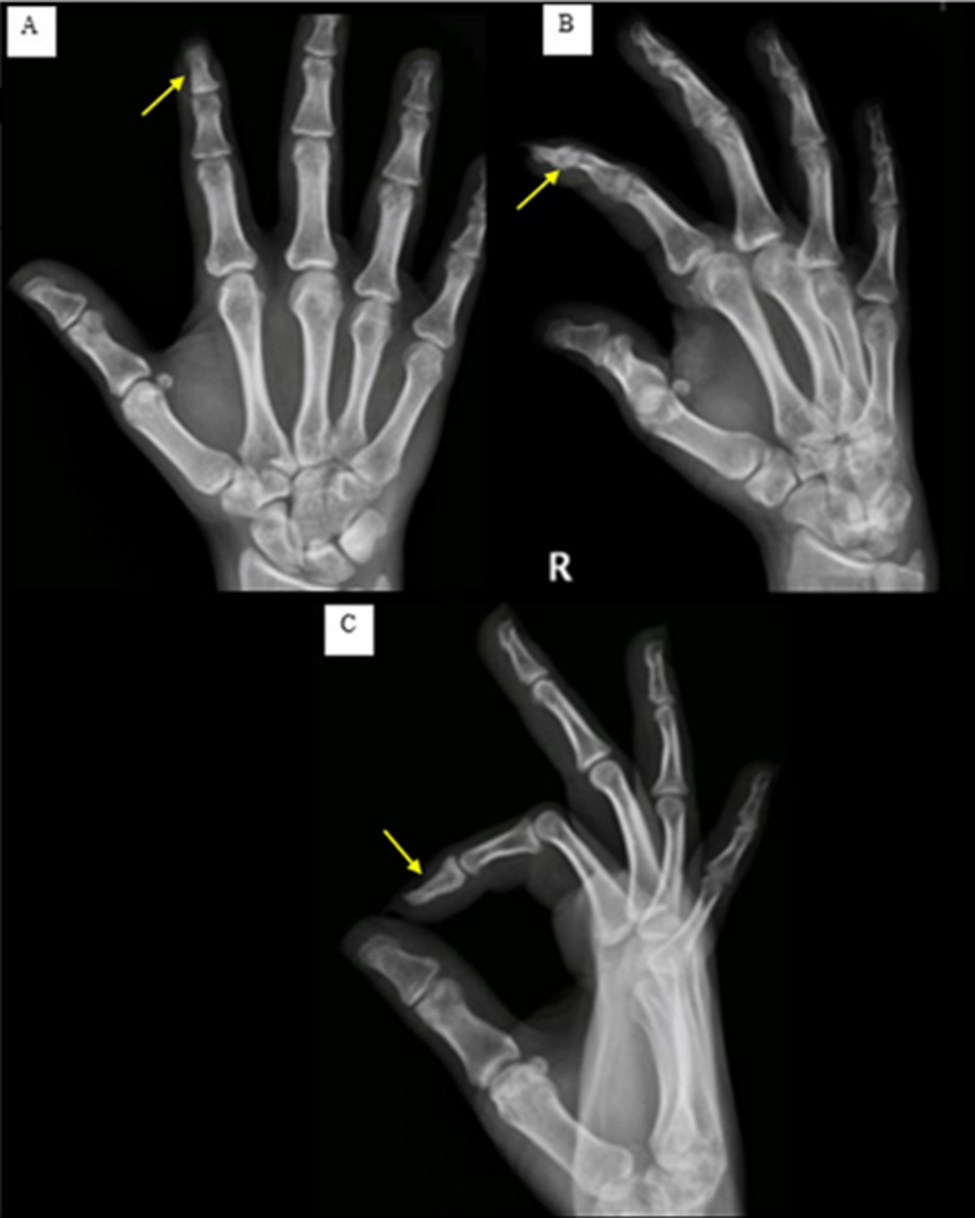
Follow-up radiographs at nine months post-operative showing a decrease in size of the cyst involving the distal phalanx of the right index finger with a relative increased sclerosis on (A) AP, (B) oblique, and (C) lateral views (yellow arrows). AP, anteroposterior.

The patient was followed up every three months and showed full clinical recovery at six months. Follow-up radiographs at three months (Figure 5) and nine months (Figure 6) were done.

## Discussion

In 1935, Jaffe introduced the term osteoid osteoma for a certain small, round tumor containing a central core referred to as a nidus [6]. High levels of prostaglandin were found in the nidus, which was thought to be the mediator of pain, especially at night that is relieved by NSAIDs. Only 10% of osteoid osteomas occur in the hands and feet [2], with the proximal phalanx being much more common than the distal phalanx, and the index finger being mostly affected [6,7]. A literature review done by Liu et al. [8] showed that out of 289 cases of osteoid osteoma involving the hand and wrist, only 19.4% (56/289) occurred in the terminal phalanx while 33.2% (96/289) occurred in the proximal phalanx; however, the middle finger was the most commonly affected site (34.7%), followed by the index finger (22.8%). According to Simon et al. [9], a retrospective study of 37 osteoid osteoma cases showed that 5.9% of the cases involved the hand with the phalanges being the most common site (59.5%), followed by the metacarpals (24.3%) and finally the carpal bones (16.2%). This finding was consistent with a literature review done by Liu et al. [8] in regard to the phalanges being the most common location with 59.2%; however, it was inconsistent when defining the frequency concerning the other two locations in which carpal bones were found to be the more common site with 30.1% compared to the metacarpal region (10.7%). The diagnostic problem regarding phalangeal osteoid osteomas was attributed to their unusual clinical, radiological, and histological characteristics. These lesions may present with painless swelling or monoarticular arthritis in addition to an uncommon lytic appearance instead of reactive sclerosis, or an unclear nidus [2].

Delay in diagnosis and, in most cases, misdiagnosis has been frequently reported with such lesion, which leads to the wrong choice of treatment initially [2]. In a study performed by Burger et al. [5], six out of seven patients were initially misdiagnosed. Diagnoses such as enchondroma, benign nerve sheath tumor, monoarticular arthritis, posttraumatic changes, osteochondroma, and infection were all made based on the clinical and radiological presentation prior to curettage and biopsy [5]. Even though the mechanism of development of osteoid osteoma is still unclear, it has been reported that 18-50% of patients with an osteoid osteoma of the wrist and hand reported a traumatic event. This made the diagnosis more difficult since it could be considered as a stress fracture or capsular strain [2, 5]. According to Meng et al. [10], the absence of reactive sclerosis was the most noticeable unusual radiological feature to be found in such lesions and was thought to be due to the poor development of the periosteum in the phalanges. Marck et al. [11] reported that monoarticular arthritis was thought to be the second most common unusual feature after the radiological absence of reactive sclerosis. Such characteristic was correlated with our patient's presentation along with the clubbing of the digit that was another additional rare characteristic mentioned in multiple studies [6]. When discussing osteoid osteoma of the phalanges, the risk of misdiagnosis is not solely restricted to surgeons or radiologists, however, because of the atypical histological features, pathologists also find difficulty reaching an accurate diagnosis. In larger bones, a clear, well-defined zone between the nidus and reactive bone is usually observed. One feature that has been specifically found in osteoid osteoma of the phalanges was the ill-defined zones where cancellous bone intermingles with nidus tissue. Other unusual findings such as loss of dense reactive sclerotic bone surrounding the nidus tissue and increased cortex diameter have been reported [5].

Regarding the management of osteoid osteoma, a variety of interventions have been described in the literature. They can range from a minimally invasive percutaneous radiofrequency ablation introduced by Rosenthal et al. [12] in 1992, to more invasive procedures such as curettage, excisional biopsy, or en bloc resection [12]. En bloc excision or curettage remains the treatment of choice for osteoid osteoma. Curettage has an increased probability of incomplete excision, thus presenting a higher potential of recurrence eventually leading to additional surgical intervention and a necessity for bone grafting to decrease the risk of postoperative fractures [13]. Even though radiofrequency ablation has been widely used nowadays for the management of osteoid osteoma, its use in the treatment of hand osteoid osteoma is limited since there is an increased iatrogenic risk to the neurovascular structure from thermal injury and the inability for histopathology examination [12].

## Conclusions

Although osteoid osteoma is a relatively frequent benign bone-forming tumor, its pathophysiology remains unclear when it comes to the hand. In the distal phalanges, osteoid osteoma can present with unusual clinical, radiological, and histological characteristics, resulting in a delay in diagnosis. Therefore, in patients presenting with any uncommon features involving the distal phalanx, it is a necessity to include osteoid osteoma in every differential diagnosis.
